# Percepção Inadequada do Risco Cardiovascular e Baixo Conhecimento sobre Hipercolesterolemia Familiar em Indivíduos com Hipercolesterolemia Grave

**DOI:** 10.36660/abc.20190516

**Published:** 2021-04-08

**Authors:** Raul D. Santos, Carolina Pereira, Fernando Cesena, Antonio Gabriele Laurinavicius, Viviane Tabone, Marcio Sommer Bittencourt

**Affiliations:** 1 Hospital Israelita Albert Einstein São PauloSP Brasil Hospital Israelita Albert Einstein , São Paulo , SP - Brasil; 2 Hospital das Clínicas Faculdade de Medicina Universidade de Sao Paulo São PauloSP Brasil Instituto do Coração (InCor) do Hospital das Clínicas da Faculdade de Medicina da Universidade de Sao Paulo , São Paulo , SP - Brasil; 3 Escola de Enfermagem Anna Nery Universidade Federal do Rio de Janeiro Rio de JaneiroRJ Brasil Escola de Enfermagem Anna Nery , Universidade Federal do Rio de Janeiro , Rio de Janeiro , RJ - Brasil

**Keywords:** Hipercolesterolemia, Fatores de Risco, Hiperlipoproteinemia Tipo II, Aterosclerose, Programas de Rastreamento

## Abstract

**Fundamento:**

Indivíduos com hipercolesterolemia grave apresentam alto risco de desenvolver doença cardiovascular aterosclerótica (DCVA). Muitos deles apresentam hipercolesterolemia familiar (HF).

**Objetivos:**

Avaliar, a partir da perspectiva dos pacientes, o nível de conhecimento sobre a hipercolesterolemia grave, especialmente em relação a HF, DCVA, percepção de risco, desempenho do rastreamento em cascata e tratamento de indivíduos participantes de um programa de avaliação periódica de saúde.

**Métodos:**

De um banco de dados de 70.000 brasileiros avaliados entre 2006 e 2016, 1.987 (2,8%) atenderam aos critérios de inclusão (idade ≥ 18 anos e LDL-C ≥ 190 mg/dL ou ≥ 160 mg/dL se sem uso de estatinas ou em terapia com estatinas, respectivamente). Desses, 200 foram aleatoriamente convidados a preencher um questionário extenso. A HF foi diagnosticada em caso de suspeita pelo médico responsável.

**Resultados:**

Embora 97% da amostra (48±9 anos; 16% do sexo feminino; 95% com ensino superior; 88% em prevenção primária; LDL-C 209±47 mg/dL) tenha apresentado hipercolesterolemia grave, apenas 18% e 29,5% se consideravam de alto risco para desenvolver DCVA e relataram saber sua meta recomendada de LDL-C, respectivamente. Em relação à possibilidade de o colesterol alto ser uma doença hereditária, 58% relataram conhecimento sobre o fato; 24,5% (n = 49) já tinham ouvido falar em HF; e apenas 14% (n = 20) foram previamente identificados com suspeita de HF (idade ao diagnóstico de HF: 35±12 anos; 79% e 31% foram diagnosticados com > 30 e > 40 anos, respectivamente). Apenas 2,5% foram submetidos a testes genéticos; 17%, à rastreamento em cascata; e 17% não faziam uso de tratamento farmacológico.

**Conclusões:**

Identificou-se uma importante lacuna na percepção de risco, no controle do colesterol e em aspectos relacionados à HF em indivíduos com hipercolesterolemia grave. (Arq Bras Cardiol. 2021; [online].ahead print, PP.0-0)

## Introdução

A hipercolesterolemia é um fator causal comprovado de doença cardiovascular aterosclerótica (DCVA). ^1^ Tanto diretrizes brasileiras quanto norte-americanas ^2 , 3^ classificam indivíduos com hipercolesterolemia grave [colesterol de lipoproteína de baixa densidade (LDL-C) > 190 mg/dL] como tendo alto risco de desenvolver DCVA, principalmente coronariopatia. Entre esses indivíduos, vários podem sofrer de hipercolesterolemia familiar (HF) heterozigótica, uma doença autossômica dominante que afeta aproximadamente 1/250 dos indivíduos em geral. ^4 , 5^ A HF é caracterizada por concentrações elevadas de LDL-C desde o nascimento e está associada a um risco de 10 a 13 vezes maior de desenvolvimento de DCVA na população em geral. ^4 , 6^ É amplamente aceito o fato de que a HF não é corretamente manejada na maioria dos países. ^7 , 8^ No entanto, os dados epidemiológicos ainda são escassos, ^9 , 10^ e as estimativas sobre prevalência, diagnóstico, tratamento e controle em diferentes partes do mundo ainda dependem predominantemente da opinião de especialistas.

Programas de avaliação periódica de saúde fornecem uma boa oportunidade para o diagnóstico de hipercolesterolemia e, consequentemente, de HF. A identificação de um caso índice pode dar início ao rastreamento em cascata, com o objetivo de identificar os membros afetados dentro de uma determinada família com HF. ^11^ No entanto, a maioria dos indivíduos com hipercolesterolemia não tem conhecimento sobre a HF, a dominância e distribuição familiar e, consequentemente, o risco alto, mas evitável de DCVA. ^12^

O objetivo do presente estudo foi avaliar o grau de conhecimento da percepção de risco em relação às DCVAs em pacientes com hipercolesterolemia grave, principalmente naqueles com suspeita de HF que participam de um programa de avaliação periódica de saúde. Em relação aos programas de avaliação, também avaliamos se medidas de cuidado de HF, como rastreamento em cascata e uso de tratamento farmacológico, foram realizadas de forma adequada, de acordo com as diretrizes de gerenciamento da doença. ^2^

## Métodos

De um banco de dados com 70.000 brasileiros submetidos a uma avaliação periódica de saúde obrigatória patrocinada pelo empregador entre 2006 e 2016 no Hospital Israelita Albert Einstein, em São Paulo, 1.987 (2,8%) atenderam aos critérios de inclusão [≥ 18 anos e LDL-C em jejum ≥ 190 mg/dL (sem uso de estatinas) ou ≥ 160 mg/dL (em terapia com estatinas)]. Desses indivíduos, 200 foram aleatoriamente convidados por telefone ou e-mail para participar do estudo ao longo de 2017. O procedimento aleatório consistiu em gerar um número de sequência aleatória, ordenar os participantes de acordo com esses números e, em seguida, chamá-los de acordo com a ordem aleatória. A amostra do estudo foi selecionada por conveniência; caso os indivíduos aceitassem participar, era obtido consentimento informado oral e realizada entrevista por telefone de acordo com um questionário estruturado desenvolvido para o presente estudo (Material Suplementar). Caso um indivíduo se recusasse a participar ou não pudesse ser contatado, o próximo da lista de randomização era convidado a participar. Este estudo foi aprovado pelo Comitê de Ética em Pesquisa do Hospital Israelita Albert Einstein.

O protocolo de avaliação de saúde foi previamente descrito e consistiu em avaliações clínicas e laboratoriais. ^13^ A pesquisa estruturada (Material Suplementar) incluiu questões sobre hipercolesterolemia, conhecimento sobre HF, diagnóstico, adesão ao tratamento, rastreamento em cascata em parentes de primeiro grau e percepção do paciente sobre o risco de DCVA. Foi considerada suspeita de HF em caso de sugestão ou diagnóstico pelo médico responsável.

### Análise estatística

Trata-se de um estudo descritivo, e a normalidade dos dados foi avaliada pelo teste de Kolmogorov-Smirnov com nível de significância de 5%. As variáveis contínuas são apresentadas como média e desvio padrão ou como mediana e quartis para variáveis que não seguem distribuição normal. As variáveis categóricas são apresentadas como contagens e proporções absolutas. A idade ao diagnóstico é apresentada em um histograma. A análise estatística foi realizada com o *software* Stata, versão 14.0 (StataCorp, EUA).

## Resultados

### Características gerais dos participantes com hipercolesterolemia grave

**Figura 1 (Ilustração Central):**
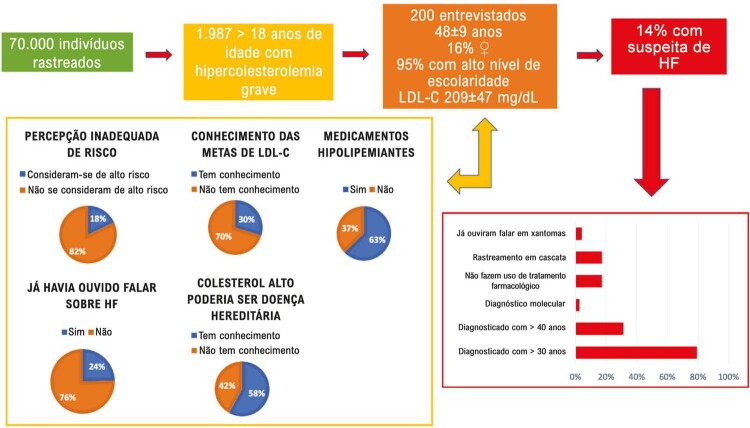
– Sumariza os principais achados do estudo em toda a amostra (n = 200) e naqueles aonde foi feita suspeita de HF (n = 29). HF: Hipercolesterolemia familiar; LDL-C: LDL-colesterol.

A Tabela 1 mostra as características clínicas e laboratoriais dos 200 participantes inscritos e dos 29 (14,5%) indivíduos com suspeita de HF. A Figura 1 (Ilustração Central) resume os resultados do estudo. No geral, a maioria dos participantes eram do sexo masculino, 95% possuíam escolaridade superior e 12% (n = 24) sofreram um evento prévio de DCVA (infarto do miocárdio, angina, revascularização do miocárdio ou acidente vascular cerebral). Ainda, 97% (n = 195) estavam cientes de que apresentavam níveis de colesterol muito altos, e 58% (n = 116) foram informados por seus médicos de que o colesterol alto poderia ser uma doença hereditária. De fato, 76% (n = 152) relataram ter um parente de primeiro grau com colesterol alto, mas apenas 4,5% (n = 9) tiveram seus parentes chamados para verificação dos níveis de colesterol no sangue e confirmação da informação.

**Tabela 1:** – Características clínicas e laboratoriais de indivíduos com hipercolesterolemia e indivíduos com suspeita de HF

	Geral (n = 200)	Suspeita de HF (n = 29)
Idade (anos)	48±9	44±9
Sexo feminino n (%)	34 (16%)	6 (23%)
Hipertensão n (%)	21 (11%)	1 (4%)
Diabetes n (%)	7 (3,5%)	0
Fumantes n (%)	26 (13%)	5 (19%)
DCVA prévia n (%)	24 (12%)	4 (14%)
Terapia hipolipemiante atual n (%)	125 (62,5%)	24 (83%)
Idade em que a terapia hipolipemiante foi iniciada (anos)	41,2 ± 9,6	36.6±11.1
Parentes de primeiro grau rastreados para colesterol alto	9 (4,5%)	5 (17%)
Colesterol total (mg/dL)	290±32	307±58
HDL-C (mg/dL)	47±13	48±13
LDL-C (mg/dL)	209±47	224±55
Triglicerídeos (mg/dL)	139 (106 – 212)	142 (97 – 232)
Glicemia (mg/dL)	95±30	87±7
HbA1c %	5.7±0.9	5.5±0.3

*Apenas estatísticas descritivas; não foi realizada nenhuma comparação formal entre os grupos devido à duplicidade de pacientes. Dados contínuos expressos em média ± desvio padrão, exceto triglicerídeos, expressos em mediana e quartis; dados categóricos expressos como frequências (%); HbA1c- hemoglobina glicosilada.*

Embora 42,5% (n = 85) tenham relatado ter um parente de primeiro grau com manifestação prévia de DCVA, apenas 19 (9,5%) lembravam de o evento ter ocorrido antes dos 55 anos. No geral, apesar dos níveis muito elevados de colesterol, apenas 18% (n = 36) se consideravam de alto risco para desenvolver DCVA, enquanto 43,5% (n = 87) acreditavam ter baixo risco durante os próximos 10 anos. Quando questionados sobre as implicações do colesterol alto para a saúde, apenas 11% (n = 22) consideravam o colesterol alto mais significante do que diabetes ou hipertensão como fator de risco para DCVA, e 71% (n = 139) consideravam o diabetes como a mais grave das três condições.

A maioria dos entrevistados compareceu a consultas médicas regulares; 72,5% (n = 145) consultaram seus médicos e 73% (n = 146) fizeram exames no último ano para determinar o nível de colesterol no sangue. No entanto, apenas 34,5% (n = 69) relataram saber o resultado do seu último teste de colesterol. Apenas 29,5% (n = 59) relataram saber sua meta de LDL-C recomendada de acordo com o estado de risco individual de DCVA. Curiosamente, desses, apenas 1 (1,7%), 9 (15%), 4 (6,8%) e 3 (5,1%) indivíduos identificaram valores de LDL-C < 70 mg/dL, < 100 mg/dL, < 130 mg/dL e < 160 mg/dL, respectivamente, como possíveis metas recomendadas de acordo com o risco. ^2 , 14^

Em relação ao uso de hipolipemiantes, 39% (n = 78) passaram por alteração dietética antes do início da terapia farmacológica e 62,6% estavam em uso desses fármacos (n = 125). Dos indivíduos que usavam medicamentos hipolipemiantes, 78% (n = 100) relataram tomar seus medicamentos diariamente, 85% (n = 110) alteraram as doses da medicação para aumentar a redução do colesterol e 15% (n = 19) relataram eventos adversos. Os motivos relatados para a interrupção dos medicamentos foram a própria decisão do paciente (54,8%), eventos adversos (22,6%), orientação médica (19,4%) e outros (3,2%).

### Indivíduos com suspeita de HF

**Figura 2:**
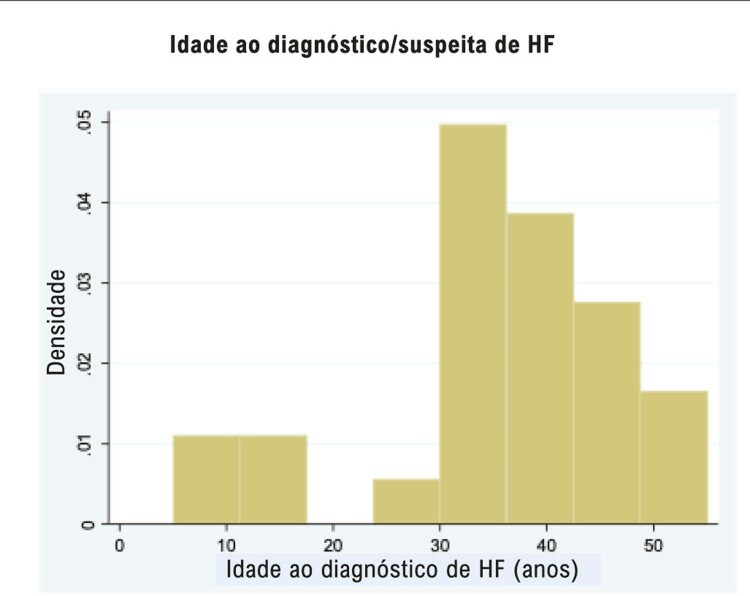
Frequência de idade ao diagnóstico/suspeita de hipercolesterolemia familiar (HF).

Apenas 24,5% (n = 49) dos participantes com hipercolesterolemia já tinham ouvido falar em HF e, desses, 29 (59%) foram previamente identificados com suspeita de HF por um profissional de saúde. A média de idade (DP) ao diagnóstico de suspeita de HF foi 35±12 anos. A Figura 2 mostra a distribuição de idade ao diagnóstico de HF; 79% e 31% foram diagnosticados após os 30 e 40 anos, respectivamente. O diagnóstico genético foi realizado em apenas 5 (17,2%) dos indivíduos com suspeita de HF, e apenas 2 (4%) já tinham ouvido falar em xantomas. É importante ressaltar que, embora 27 (93%) indivíduos com suspeita de HF tenham relatado ter sido informados de que outros familiares poderiam ter essa doença, apenas 5 (17%) lembravam de seus parentes terem sido chamados para verificação do nível de colesterol no sangue. O tratamento foi iniciado, em média, após os 35 anos ( Tabela 1 , e 17% (n = 5) dos indivíduos com suspeita de HF não faziam uso de terapia hipolipemiante farmacológica.

## Discussão

Não existem dados da população brasileira em relação ao conhecimento dos pacientes sobre as implicações da hipercolesterolemia, principalmente das formas graves como a HF. A maioria dos estudos conduzidos até o momento avaliou o conhecimento geral sobre o diagnóstico de hipercolesterolemia e não o conhecimento específico sobre as formas graves e suas consequências. ^15 , 16^ Esta pesquisa, realizada em uma população de alta escolaridade, predominantemente do sexo masculino, com hipercolesterolemia grave e frequentadora de um programa de avaliação de saúde em São Paulo, sugere que o conhecimento das implicações do colesterol muito alto e, principalmente, da HF e seus aspectos relacionados é baixo.

Mais impressionantes foram os achados relacionados à percepção inadequada ou falta de conhecimento dos pacientes sobre o alto risco associado à hipercolesterolemia grave, já que apenas um em cada cinco indivíduos reconheceu estar em alto risco de desenvolver DCVA, apesar de as diretrizes médicas declararem o contrário. ^2 , 3 , 14^ Além disso, houve falta de conhecimento por parte dos indivíduos sobre as metas de LDL-C recomendadas para seu nível de risco e o não uso de tratamento farmacológico em quase 40% dos participantes do estudo. Outro achado preocupante foi que, entre aqueles que pararam de tomar a medicação, quase 75% o fizeram por decisão pessoal ou orientação médica e não pela ocorrência de eventos adversos. Uma possível explicação para esses achados é que apenas um em cada 10 participantes do estudo considerou o colesterol alto como o fator de risco mais importante em comparação a diabetes e hipertensão. Apesar do papel desempenhado pela hipertensão, não há dúvidas sobre o papel central e causal da hipercolesterolemia e do consequente aumento do risco, atribuído às formas graves, principalmente à HF, de coronariopatia. ^1 , 17 , 18^ Esses achados sugerem a necessidade de melhora no conhecimento sobre o papel desempenhado pelo colesterol na DCVA. Como mostrado anteriormente, a falta de conhecimento sobre doenças crônicas como a hipercolesterolemia está associada ao uso inadequado de tratamento farmacológico em países de baixa renda, onde os custos dos medicamentos têm implicações importantes _._^19 , 20^ Isso se torna ainda mais preocupante ao considerar o elevado nível social e educacional dos participantes do estudo.

A HF é gravemente subdiagnosticada e subtratada, ^4 , 7^ e o diagnóstico (geralmente com > 40 anos) ^21^ e consequente tratamento tardios estão associados a taxas elevadas de coronariopatia, conforme observado em casos índices no Brasil ^22^ e em outros países. ^10^ Há evidências de que, mesmo em indivíduos com hipercolesterolemia grave, ou seja, LDL-C > 190 mg/dL, a presença de um defeito genético autossômico dominante implica em um risco relativo de DCVA 4 vezes maior. ^17^ Considerando o traço autossômico dominante da HF, um modelo adequado de cuidado para a doença inclui não apenas a identificação e o tratamento de casos índices, mas o rastreamento em cascata de parentes afetados. ^7^

Este estudo sugere que há um baixo nível de conhecimento sobre a HF entre os indivíduos com hipercolesterolemia grave, pois apenas um em cada quatro participantes do estudo relatou conhecimento sobre a doença. Isso ocorre apesar de uma alta prevalência relatada de colesterol elevado em parentes de primeiro grau. Além disso, naqueles com suspeita de HF, a doença foi diagnosticada de forma tardia, o que provavelmente explica a elevada frequência de DCVA na população.

A indicação de rastreamento em cascata pelos médicos responsáveis foi muito baixa, e quase 20% dos pacientes com suspeita de HF não faziam uso de terapia farmacológica. Esses achados não diferem muito de um estudo recente de indivíduos submetidos a triagem em cascata molecular por suspeita de HF em um centro terciário no Brasil. ^23^ No estudo realizado por Souto et al., apenas 20% dos casos índice ou parentes de primeiro grau participantes do programa de rastreamento em cascata relataram suspeita prévia de diagnóstico de HF, enquanto 71% faziam uso de tratamento farmacológico hipolipemiante.

No *Cascade Screening for Awareness and Detection* (CASCADE) do registro de HF dos EUA, ^24^ houve um intervalo médio de 6 anos entre o diagnóstico de hipercolesterolemia e o início do tratamento hipolipemiante e subsequente diagnóstico de HF. Esses resultados são compatíveis com os achados do presente estudo, no qual foi diagnosticada hipercolesterolemia grave; o tratamento farmacológico foi sugerido/iniciado na maioria dos participantes do estudo, mas apenas 1/4 já havia sido informado sobre HF por seu médico. Nossos resultados sugerem uma lacuna importante no conhecimento sobre HF não apenas entre os pacientes, mas também entre os médicos. De fato, um conhecimento insuficiente sobre a HF entre médicos ^25 - 27^ ou pacientes ^28^ tem sido relatado em diferentes partes do mundo, inclusive no Brasil.

As limitações deste estudo incluem a amostra relativamente pequena (no entanto, é importante ressaltar que o LDL-C > 190 mg/dL costuma afetar cerca de 5% da população); e o fato de o desenho transversal do estudo ter mostrado apenas associações, não tendo sido realizada uma investigação formal das causas dos nossos achados. Além disso, as características específicas da população, principalmente a alta escolaridade, não permitem que os resultados sejam extrapolados para a população brasileira de menor escolaridade, mas podem sugerir que achados mais graves possam ser encontrados. Por último, não realizamos comparação direta da percepção e do manejo de risco entre os indivíduos com suspeita ou não de HF; e, embora os participantes tenham sido ativamente questionados, os resultados estão sujeitos a viés de memória. De qualquer forma, os resultados são notáveis e compatíveis com outros estudos, ^24 - 28^ além de mostrarem uma importante necessidade não atendida de educação sobre a importância da hipercolesterolemia grave e, especificamente, da HF.

## Conclusões

Identificou-se uma lacuna importante na percepção de risco, no controle do colesterol e em aspectos relacionados à HF em indivíduos com hipercolesterolemia grave. Investigações adicionais e mais amplas são necessárias para confirmar os resultados, e o desenvolvimento de programas de educação para pacientes e médicos é necessário para o preenchimento dessa lacuna de conhecimento.
