# Supplementary material for: Percepção Inadequada do Risco Cardiovascular e Baixo Conhecimento sobre Hipercolesterolemia Familiar em Indivíduos com Hipercolesterolemia Grave
Source: Arq Bras Cardiol. 2021 Apr 8;116(4):706–12. [Article in Portuguese] doi: 10.36660/abc.20190516 (PMC8121404; doi:10.36660/abc.20190516)
Supplement: Translation

# Translation: Cardiovascular Risk Misperception and Low Awareness of Familial Hypercholesterolemia in Individuals with Severe Hypercholesterolemia

**Authors:** Raul D. Santos, Carolina Pereira, Fernando Cesena, Antonio Gabriele Laurinavicius, Viviane Tabone, Marcio Sommer Bittencourt

**Affiliations:** 1 Brazil Hospital Israelita Albert Einstein, São Paulo, SP - Brazil; 2 Brazil Instituto do Coração (InCor) do Hospital das Clínicas da Faculdade de Medicina da Universidade de Sao Paulo, São Paulo, SP - Brazil; 3 Brazil Escola de Enfermagem Anna Nery, Universidade Federal do Rio de Janeiro, Rio de Janeiro, RJ - Brazil

**Keywords:** Hypercholesterolemia Risk Factors, Hyperlipoproteinemia Type II, Atherosclerosis, Mass Screening

## Abstract

**Background:**

Individuals with severe hypercholesterolemia are at a high risk of developing atherosclerotic cardiovascular disease (ASCVD). Many of them have familial hypercholesterolemia (FH).

**Objectives:**

To assess from a patient perspective the degree of awareness about severe hypercholesterolemia, especially FH, ASCVD risk perception, cascade screening performance, and treatment of individuals participating in a routine health evaluation program.

**Methods:**

From a database of 70,000 Brazilian individuals evaluated between 2006 and 2016, 1,987 (2.8%) met the inclusion criteria (age ≥ 18 years and LDL-C ≥ 190 mg/dL or ≥ 160 mg/dL, respectively, if not in use of statins or on statin therapy). Two-hundred individuals were randomly invited to complete an extensive questionnaire. FH was diagnosed if suspected by the attending physician.

**Results:**

Although 97% of the sample (age 48±9 years; 16% women; 95% college/university education; 88% primary prevention; LDL-C 209±47 mg/dL) had severe hypercholesterolemia, only 18% and 29.5% believed to be at high ASCVD risk and reported knowledge of their recommended LDL-C goal, respectively. Fifty-eight percent reported being informed that high cholesterol could be a family disease, 24.5% (n = 49) had ever heard about FH, and merely 14% (n = 29) had been previously identified as suspected of having FH (age at FH diagnosis 35±12 years; 79% and 31% diagnosed, respectively, > 30 and > 40 years old). Only 2.5% underwent genetic tests, 17% underwent cascade screening, and 17% were not in use of pharmacological treatment.

**Conclusions:**

An important gap in risk perception, cholesterol management, and aspects related to FH was encountered in individuals with severe hypercholesterolemia. (Arq Bras Cardiol. 2021; [online].ahead print, PP.0-0)

## Introduction

Hypercholesterolemia is a proven causal factor of atherosclerotic cardiovascular disease (ASCVD).^1^Both Brazilian and US guidelines^2 , 3^classify individuals with severe hypercholesterolemia (low-density lipoprotein cholesterol - LDL-C > 190 mg/dL) as being at a high risk of developing ASCVD, especially coronary heart disease. Among these, many individuals may suffer from heterozygous familial hypercholesterolemia (FH), an autosomal dominant disease affecting approximately 1/250 individuals in general.^4 , 5^FH is characterized by elevated LDL-C concentrations since birth and is associated with a 10-13-fold higher risk of ASCVD onset in the general population.^4 , 6^It is widely accepted that FH is currently mishandled in most countries.^7 , 8^However, epidemiologic data are still scarce,^9 , 10^and estimations on prevalence, diagnosis, treatment, and control in different parts of the world continue to rely predominantly on experts’ opinion.

Routine health evaluation programs provide a good opportunity to diagnose hypercholesterolemia and, consequently, FH. The identification of an index case can start cascade screening with the aim of identifying affected members within a given FH family.^11^However, most hypercholesterolemic individuals are unaware of FH, family dominance and distribution, and consequent yet preventable high ASCVD risk.^12^

The aim of the present study was to assess the degree of awareness of ASCVD risk in patients with severe hypercholesterolemia, especially in those suspected of having FH participating in a routine health evaluation program. On the latter we also evaluated if measures of care in FH such as cascade screening and use of pharmacological treatment were adequately performed according to disease management guidelines.^2^

## Methods

From a database of 70,000 Brazilian individuals undergoing a mandatory employer-sponsored routine health evaluation between 2006 and 2016 at the Hospital Israelita Albert Einstein in São Paulo, Brazil, 1,987 (2.8%) met the inclusion criteria (age ≥ 18 years and fasting LDL-C ≥ 190 mg/dL without statins or ≥ 160 mg/dL if on statin therapy). Of these, 200 individuals were randomly invited by phone or e-mail to participate in the study during 2017. The random procedure consisted of generating a random sequence number, ordering participants by those numbers, and then calling them following the random order. The study sample was selected by convenience; if subjects accepted to participate, an oral informed consent was obtained and an interview was performed by telephone according to a structured questionnaire developed for the present study (Supplemental Material). If the individual refused to participate or could not be contacted, the next on the randomization list was invited to participate. This study was approved by the Ethics Committee of Hospital Israelita Albert Einstein.

The health evaluation protocol was previously described and consisted of clinical and laboratory evaluations.^13^The structured survey (Supplementary Material) included questions about hypercholesterolemia, FH awareness, diagnosis, adherence to treatment, cascade screening in first-degree relatives, and ASCVD risk perception from a patient perspective. FH was considered suspected if the attending physician suggested or made this diagnosis.

### Statistical Analysis

This is a descriptive study, and data normality was assessed using the Kolmogorov-Smirnov test with a significance level of 5%. Continuous variables are presented as mean and standard deviation or as median and quartiles for variables known not to be normally distributed. Categorical variables are presented as absolute counts and proportions. Age at diagnosis is presented in a histogram. Statistical analysis was performed using Stata version 14.0 (StataCorp, USA).

## Results

### General characteristics of participants with severe hypercholesterolemia

Table 1 shows clinical and laboratory characteristics of the 200 enrolled participants and the 29 (14.5%) individuals in which FH was suspected. Figure 1 (Central Illustration) summarizes the study results. Overall, most individuals were men, 95% had college/university degree, and 12% (n= 24) had suffered a previous ASCVD event (myocardial infarction, angina, myocardial revascularization, or stroke). Ninety-seven percent (n = 195) were aware of having very high cholesterol levels and 58% (n = 116) had been informed by their physicians that high cholesterol could be a family disease. Indeed, 76% (n = 152) reported having a first-degree relative with high cholesterol. However, only 4.5% (n = 9) had their first-degree relatives invited to test their blood cholesterol levels and confirm that information.

**Table 1:** – Clinical and laboratory characteristics of hypercholesterolemic individuals and of those suspected of FH

	General (n = 200)	Suspected FH (n = 29)
Age (years)	48±9	44±9
Female sex n (%)	34 (16%)	6 (23%)
Hypertension n (%)	21 (11%)	1 (4%)
Diabetes n (%)	7 (3.5%)	0
Smokers n (%)	26 (13%)	5 (19%)
Previous ASCVD n (%)	24 (12%)	4 (14%)
Current lipid-lowering therapy n (%)	125 (62.5%)	24 (83%)
Age lipid-lowering therapy was started (years)	41.2 ± 9.6	36.6±11.1
First-degree relatives screened for high cholesterol	9 (4.5%)	5 (17%)
Total cholesterol (mg/dL)	290±32	307±58
HDL-C (mg/dL)	47±13	48±13
LDL-C (mg/dL)	209±47	224±55
Triglycerides (mg/dL)	139 (106 – 212)	142 (97 – 232)
Glucose (mg/dL)	95±30	87±7
HbA1c %	5.7±0.9	5.5±0.3

*Descriptive statistics only; no formal comparison was made between the groups because of patient duplicity. Continuous data expressed as mean ± standard deviation, except for triglycerides, expressed as median and quartiles; categorical data expressed as frequencies (%). ASCVD: atherosclerotic cardiovascular disease; FH: familial hypercholesterolemia; HbA1c: glycated hemoglobin; HDL-C: high-density lipoprotein cholesterol; LDL-C: low-density lipoprotein cholesterol.*

**Figure 1:** – Central Illustration summarizes main study results in the entire study population (n = 200) and in those suspected of FH (n = 29). FH: familial hypercholesterolemia; LDL-C: low-density lipoprotein cholesterol.

Although 42.5% (n = 85) reported having a first-degree relative with a previous manifestation of ASCVD, only 19 (9.5%) recalled such event occurring before the age of 55 years. Overall, despite very high cholesterol levels, only 18% (n = 36) considered themselves as being at high ASCVD risk, while 43.5% (n = 87) believed to be at low risk for the next 10 years. When asked about the health implications of having high cholesterol, only 11% (n = 22) considered high cholesterol more important than diabetes or hypertension as risk factors for ASCVD, while 71% (n = 139) considered diabetes as the most severe of the three conditions.

Most interviewed individuals attended regular medical consultations; 72.5% (n = 145) consulted their physicians and 73% (n = 146) had their cholesterol levels determined in the past year. However, only 34.5% (n = 69) reported knowing their last cholesterol test results. Only 29.5% (n = 59) reported knowledge about their recommended LDL-C goal according to individual ASCVD risk status. Interestingly, of those, only 1 (1.7%), 9 (15%), 4 (6.8%), and 3 (5.1%) individuals identified LDL-C values < 70 mg/dL, < 100 mg/dL, < 130 mg/dL, and < 160 mg/ dL as possible recommended goals according to risk, respectively.^2 , 14^

Thirty-nine percent (n = 78) underwent a dietary change before pharmacological lipid-lowering therapy was initiated, and the therapy was being used by 62.6% (n = 125). Of those using lipid-lowering medications, 78% (n = 100) reported taking their medications on a daily basis. Eighty-five percent (n = 110) had changed medication doses to further increase cholesterol lowering, while 15% (n = 19) reported adverse events. Reported reasons for stopping medications were patients’ own decision (54.8%), adverse events (22.6%), medical orientation (19.4%), and others (3.2%).

### Individuals with suspected FH

Only 24.5% (n = 49) of hypercholesterolemic subjects had ever heard about FH and, of those, 29 (59%) had been previously identified as suspected of having FH by their healthcare providers. Mean age (SD) when suspected FH was diagnosed was 35±12 years. Figure 2 shows the distribution of age when FH was diagnosed; 79% and 31% were diagnosed, respectively, after the age of 30 and 40 years. Genetic diagnosis was performed only in 5 (17.2%) of those suspected of having FH, and only 2 individuals (4%) had ever heard about xanthomas. Importantly, although 27 (93%) individuals with suspected FH reported having been told that other family members could have this disease, only 5 (17%) recalled having their relatives invited to test their blood cholesterol. Treatment was started on average after the age of 35 years ( Table 1 ), and 17% (n = 5) of those suspected of having FH were not in use of pharmacological lipid-lowering therapy.

**Figure 2:** – Frequency of age when FH was suspected/diagnosed. FH: familial hypercholesterolemia.

## Discussion

There are no data for the Brazilian population on patient awareness of implications of hypercholesterolemia, especially severe forms such as FH. Most studies thus far evaluated overall awareness of hypercholesterolemia diagnosis and not specific knowledge of the severe forms and their consequences.^15 , 16^This survey, performed in a highly educated, predominantly male population with severe hypercholesterolemia attending a health evaluation program in São Paulo, suggests that awareness of implications of very high blood cholesterol and, especially, FH and its related aspects is low.

Most striking were the findings of patient misperception or lack of knowledge about the high risk associated with severe hypercholesterolemia, as only one in five individuals recognized being at high ASCVD risk, despite medical guideline recommendations stating otherwise.^2 , 3 , 14^In addition, there was a lack of knowledge regarding recommended LDL-C goals for their level of risk and no use of pharmacological treatment in almost 40% of the study participants. Another concerning finding was that, among those who stopped their medications, almost 75% did so by their personal decision or medical orientation rather than occurrence of adverse events. One possible explanation for these findings is that only one in 10 of the study participants considered high cholesterol as the most important risk factor in comparison with diabetes and hypertension. Despite the role played by the latter, there is no doubt about the central and causal role of hypercholesterolemia and consequent elevated risk attributed to the severe forms, especially FH, in coronary heart disease.^1 , 17 , 18^These findings suggest the need for improvement in literacy about the role played by cholesterol in ASCVD. As previously shown, lack of literacy about chronic diseases such as hypercholesterolemia is associated with inadequate use of pharmacological treatment in low-income countries, where medication costs have important implications.^19 , 20^This is even more concerning considering the elevated social and educational level of the study participants.

FH is severely underdiagnosed and undertreated,^4 , 7^and late diagnosis (usually > 40 years old)^21^and consequent late treatment are associated with elevated rates of coronary heart disease, as seen in index cases in Brazil^22^and other countries.^10^Indeed, there is evidence that even in individuals with severe hypercholesterolemia, i.e., LDL-C > 190 mg/dL, the presence of an autosomal dominant genetic defect implicates in a 4-fold greater relative risk of ASCVD.^17^Considering the autosomal dominant trait of FH, an adequate model of care for this disease includes not only identification and treatment of index cases but cascade screening for affected relatives.^7^

This study suggests that there is a low level of FH awareness amongst individuals with severe hypercholesterolemia, as only 1 in 4 study participants reported knowledge about the disease. This occurs despite a high reported prevalence of elevated cholesterol in first-degree relatives. Moreover, in those with suspected FH, the disease was diagnosed late, which probably explains the elevated frequency of ASCVD in the population.

There was a very low indication for cascade screening by attending physicians, and almost 20% of patients with suspected FH were not in use of pharmacological therapy. These findings do not differ much from those of a recent study of individuals undergoing molecular cascade screening due to FH suspicion in a tertiary center in Brazil.^23^In the study conducted by Souto et al., only 20% of either index cases or first-degree relatives participating in the cascade screening program reported a previous suspicion of FH diagnosis, while 71% where in use of pharmacological lipid-lowering treatment.

In the US Cascade Screening for Awareness and Detection (CASCADE) of FH registry,^24^there was a median 6-year gap between diagnosis of hypercholesterolemia and start of lipid-lowering treatment and subsequent FH diagnosis. These results are compatible with the findings of the current study, in which severe hypercholesterolemia was diagnosed; pharmacological treatment was suggested/started in most study participants, but only one quarter had ever heard about FH from their physicians. Our results suggest an important gap in FH literacy not only among patients but also among physicians. Indeed, a poor knowledge about FH amongst either physicians^25 - 27^or patients^28^has been reported in different parts of the world, including Brazil.

Limitations of this study include the relatively small sample, but it is worth noting that LDL-C > 190 mg/dL usually affects around 5% of the population; the cross-sectional design shows only associations and there was no formal investigation of the causes of our findings; the specific characteristics of the population, especially high educational level, does not allow that results to be extrapolated for the overall Brazilian population with lower educational level, but it may suggest that more severe findings may be encountered; a direct comparison of risk perception and management between those suspected or not of FH was not performed; finally, although study participants were actively questioned, results are subject to recall bias. Nonetheless, findings are remarkable and compatible with other investigations^24 - 28^and show an important unmet need for education about the importance of severe hypercholesterolemia and, specifically, FH.

## Conclusions

An important gap in risk perception, cholesterol management, and aspects related to FH was encountered in individuals with severe hypercholesterolemia. Further and broader investigations are necessary to confirm the results, and development of education programs for both patients and physicians will be required to close this knowledge gap.
